# Tumor budding for predicting prognosis of resected rectum cancer after neoadjuvant treatment

**DOI:** 10.1186/s12957-019-1588-6

**Published:** 2019-03-14

**Authors:** Atakan Demir, Ozkan Alan, Ertugrul Oruc

**Affiliations:** 1 Division of Medical Oncology, Acibadem University School of Medicine, Acibadem Maslak Hospital, Buyukdere Cad. No: 40, 34457 Maslak, Istanbul Turkey; 2 Division of Medical Oncology, Department of Internal Medicine, Marmara University Faculty of Medicine, Pendik Education and Research Hospital, Marmara University, Fevzi Cakmak Mahallesi, Muhsin Yazicioğlu Cd No:10, 34899 Pendik, Istanbul Turkey; 3 Division of Pathology, Istanbul Tuzla State Hospital, Içmeler Mah. Piri Reis Cad. No:74, Tuzla, Istanbul Turkey

**Keywords:** Tumor budding, Prognosis, Rectum cancer

## Abstract

**Background:**

Rectum cancer is a type of colorectal cancer. Its etiology and etiopathogenesis are similar to other colon diseases. We aimed to evaluate the tumor budding for predicting prognosis of resected rectum cancer patients.

**Methods:**

We retrospectively collected the data of 75 operated rectum adenocarcinoma patients who were treated neoadjuvant chemoradiotherapy between 2013 and 2018 in Umraniye Research and Training Hospital and Acıbadem University Medical Oncology Outpatient Clinic. Tumor budding was investigated as a prognostic factor for disease-free survival.

**Results:**

This study included 75 rectum cancer patients and 51 were male (68%). Median age was 56 (range 19 to 77 years). There were 29 (39%) and 46 (61%) patients in tumor budding low-intermediate and high groups respectively. In multivariate analysis, tumor budding was found to be an independent prognostic factor for disease-free survival (*p* = 0.00).

**Conclusions:**

According to our study, having high tumor budding suggests a high likelihood of relapse. Therefore, we might need additional follow-up protocol in these patients.

## Background

Colorectal cancer (CRC) is one of the most common cancers worldwide. It is the third most frequently diagnosed cancer in men and second in women. Several trials have shown the relationship between tumor budding and disease prognosis in colorectal cancer. The aim of our study was to investigate the prognostic value of tumor budding in patients with rectum cancer who underwent radical curative surgery after neoadjuvant chemoradiotherapy.

## Introduction

Colorectal cancer (CRC) is one of the most common cancers worldwide. It is the third most frequently diagnosed cancer in men and second in women [1]. Although CRC mortality has been rapidly declining since 1990, nowadays, its rate is approximately 1.7 to 1.9% per year [2]. Neoadjuvant chemoradiotherapy is the standard therapeutic approach in rectal cancer [3, 4]. There is no ideal marker for predicting prognosis after chemoradiotherapy. Factors affecting prognosis in colorectal cancers can be summarized as individual (age, sex, family history), clinical, biochemical, pathologic prognostic factors, tumor progression at the time of diagnosis, and adjunctive therapy (surgery, adjuvant and/or neoadjuvant treatment) [5, 6]. We know that about 25% of patients with early-stage colorectal cancer develop distant metastases [7]. The TNM staging system may not be a good candidate for a prognostic parameter in colorectal cancer because some patients in the same pathological stage may present various oncological outcomes such as early recurrence or mortality [8]. Several other novel histopathological parameters are also being explored as potential prognostic biomarkers for colorectal cancer, such as tumor budding (TB), poorly differentiated clusters (PDCs), extramural vascular (vein) invasion (EMVI), perineural invasion (PNI), tumor deposits (TDs), mucin pools (MPs), and extranodal extension of nodal metastasis (ENE), but some of these are yet to be fully investigated in larger phase trials for their association with prognosis of colorectal cancer. For example, ENE has been reported to be associated with a significantly increased risk of recurrence and mortality in a meta-analysis [9, 10].

Tumor budding was defined as the presence of isolated single cells or small cell clusters of less than five cells in the literature. Tumor buddings are disturbed within the stroma at the tumor margin. They tend to lose adhesion and dissociate, and this situation causes the tumor to be aggressive. There is a close relationship between tumor budding and the process of epithelial-mesenchymal transition. In this transitional process, epithelial cells lose intracellular and cell-matrix contacts mediated by E-cadherin, resulting in invasion and ultimately metastatic cancer spread [11–13]. Several trials have shown the relationship between tumor budding and disease prognosis in colorectal cancer. Especially, tumor budding might be closely related to poor survival and high risk of recurrence [14, 15].

The aim of our study was to investigate the prognostic value of tumor budding in patients with rectum cancer who underwent radical curative surgery after neoadjuvant chemoradiotherapy.

## Method

### Patients

We retrospectively collected the data of 80 operated rectum adenocarcinoma patients who were treated with neoadjuvant chemoradiotherapy between 2013 and 2018 in Umraniye Research and Training Hospital and Acıbadem University Medical Oncology Outpatient Clinic. Inclusion criteria were histological diagnosis of non-metastatic rectal adenocarcinoma, treated with neoadjuvant chemoradiotherapy, and having complete medical records. All patients were older than 18 years old. Five patients with a complete response were excluded from the study. A total of 75 patients were evaluated.

All patients received neoadjuvant chemoradiotherapy. Radiotherapy was given for a total of 45 Gy/28 days. Capecitabine 825 mg/m^2^/day or 5-fluorouracil 200 mg/m2 D1-5 weekly was administered. All of the patients were operated on an average of 8–12 weeks.

We defined the follow-up duration as the time from the start of neoadjuvant chemoradiotherapy treatment until death of any reason/the last visit. Disease-free survival was defined as the time from date of surgery until radiological progression or death/the last visit. The data cutoff date was accepted on September 2018.

### Pathological evaluation

Seventy-five patients’ pathology slides from the adenocarcinoma area are kept in department storage and were evaluated for tumor budding in a light microscope. All rectum specimens were sliced transversely at 3–4-mm intervals, and at least eight tumor samples were taken in every specimen. However, patients’ paraffin-embedded tissue blocks were not cut into any section for hematoxylin and eosin and/or other histochemical or immunohistochemical staining.

A three-tier system, which is recommended by the ITBCC (The International Tumor Budding Consensus Conference) 2016 group, was used [16]. The ITBCC group also recommends that, in addition to the Bd category, the absolute bud count should be provided (e.g., Bd 3 (count 17)). We grouped the patients to be low-intermediate and high due to the small number of patients.

The three-tier system is categorized as:

- 0–4 buds—low budding (Bd 1)
- 5–9 buds—intermediate budding (Bd 2)
- 10 or more buds—high budding (Bd 3)

Tumor budding was assessed in one hotspot (in a field measuring 0.785 mm^2^) at the invasive front. Firstly, we selected the H&E slide with the greatest degree of budding at the invasive front and then scanned ten individual fields at medium power (10× objective) to identify the hotspot area. Ultimately, we counted tumor buds in the selected hotspot area (20× objective) and divided the bud count by the normalization factor to determine the tumor bud count per 0.7 85mm^2^ as defined by the ITBCC group.

The tumor grading was categorized into well differentiated (> 95% gland formation), moderately differentiated (50–95% gland formation), and poorly differentiated (< 50% gland formation). Patients were grouped into four categories according to the tumor-node-metastasis (TNM) staging, based on the American Joint Cancer Committee (AJCC) cancer staging manual 7th edition.

Tumor regression was assessed by the four-tier AJCC/CAP tumor regression grading system. It is categorized as [17]:

- No viable cancer cells—0 (complete response)
- Single cells or small groups of cancer cells—1 (moderate response)
- Residual cancer outgrown by fibrosis—2 (minimal response)
- Minimal or no tumor kill, extensive residual cancer—3 (poor response)

### Statistical analysis

Disease-free survival (DFS) was calculated using the Kaplan-Meier method from the operated date. Prognostic factors were compared using the log-rank test in univariate analysis. Hazard ratios (HR) with 95% confidence intervals (CI) were also calculated. All *p* values were two-sided in the tests, and *p* values of 0.05 were considered statistically significant. Multivariate analysis was carried out using the Cox proportional hazards model to assess the effect of prognostic factors on survival.

## Results

### Patients demographic and clinical characteristics outcomes

Data from a total of 75 operated rectum cancer patients treated with systemic treatment and available medical records were analyzed retrospectively. Fifty-one of the seventy-five patients were male (68%) and the median age was 56 (range 19–77 years). Pretreatment demographic and clinical characteristics of patients based on tumor budding groups for the entire study cohort were outlined in Table 1. There were 29 (39%) and 46 (61%) patients in tumor budding low-intermediate and high groups respectively. Baseline demographics and disease characteristics were similar between the two groups, with the exception of microsatellite instability. Tumor budding high group had more microsatellite instability patients compared to the low-intermediate group (*p* = 0.02).

**Table 1:** Demographic and clinicopathologic findings

		All patients, *n* = 75	Tumor budding		*p*
			Low-intermediate*, n* = 29	High*, n* = 46	
Gender, *N* (%)	Female	24 (32)	9 (31)	15 (32)	0.8
	Male	51 (68)	20 (69)	31 (68)	
Tumor localization, *N* (%)	Proximal	13 (17)	7 (24)	6 (13)	0.4
	Middle	32 (43)	11 (38)	21 (46)	
	Distal	30 (40)	11 (38)	19 (41)	
Pathology, *N* (%)	Adenocarcinoma	70 (94)	26 (90)	44 (96)	0.3
	Mucinous adenocarcinoma	5 (6)	3 (10)	2 (4)	
Neoadjuvant chemotherapy, *N* (%)	5-Flouracil	30 (40)	11 (38)	19 (41)	0.5
	Capecitabine	45 (60)	18 (62)	27 (69)	
Surgery, *N* (%)	Anterior	4 (5)	2 (6)	2 (4)	0.5
	Low anterior	40 (55)	17 (63)	23 (52)	
	Very low anterior	7 (10)	1 (3)	6 (13)	
	Miles	23 (30)	9 (28)	14 (29)	
	Total colectomy	1 (1)	0	1 (2)	
Total lymph node excision (median) (min–max)		18 (5–32)	19 (5–27)	17 (5–32)	0.3
Pathology stage, *N* (%)	1	14 (19)	7 (24)	7 (15)	0.5
	2	25 (33)	10 (34)	15 (33)	
	3	36 (48)	12 (42)	24 (52)	
Tumor regression	Moderate response	14 (19)	7 (24)	7 (15)	0.4
	Minimal response	23 (30)	10 (34)	13 (28)	
	Poor response	38 (51)	12 (42)	26 (57)	
Grade, *N* (%)	Well	45 (60)	18 (62)	27 (59)	0.9
	İntermediate	10 (13)	3 (10)	7 (15)	
	Poorly	20 (27)	8 (28)	12 (26)	
Microsatellite instability status, *N* (%)	Low	48 (64)	23 (79)	25 (54)	0.02
	High	27 (36)	6 (21)	21 (46)	

### Survival outcomes

**Fig. 1:**
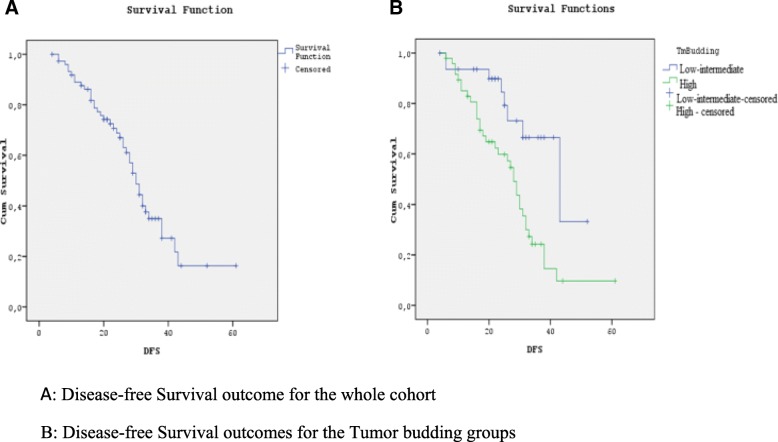
**a**, **b** Disease-free survival graphic by Kaplan-Meier

Adjuvant treatment, relapse, mutational status characteristics, and overall clinical outcomes are shown in Table 2. Median follow-up duration was 35 months (range 9–65 months). During the follow-up period, 41(55%) of the 75 patients relapsed. For the whole cohort, median disease-free survival (DFS) was 30 months (95% CI 26.8–33.1) (Fig. 1a). According to the tumor budding low-intermediate and high groups, median DFS was 43 months in the tumor budding low-intermediate group (95% CI 25.7–60.2) and 28 months in the high group (95% CI 25.2–30.7) (*p* = 0.003) (Fig. 1b).

**Table 2:** Treatment characteristics and clinical outcomes

Characteristics		All patients, *n* = 75	Tumor budding		*p*
			Low-intermediate, *n* = 29	High, *n* = 46	
Adjuvant chemotherapy, *N* (%)	FUFA	11 (14)	2 (6)	9 (19)	0.2
	Capecitabine	8 (10)	5 (17)	3 (6)	
	Folfox	23 (30)	8 (28)	15 (33)	
	Xelox	36 (46)	14 (49)	19 (42)	
Relapse, *N* (%)	Yes	41 (55)	8 (28)	33 (72)	0.00
	No	34 (45)	21 (72)	13 (28)	
Relapse pattern, *N* (%)	Local	17 (41)	4 (50)	13 (39)	0.9
	Visceral	24 (59)	4 (50)	20 (61)	
KRAS status*, N* = 41 (%)	Wild	9 (22)	3 (38)	6 (18)	0.3
	Mutant	32 (78)	5 (62)	27 (82)	
NRAS status*, N* = 9 (%)	Wild	8 (89)	2 (67)	6 (100)	0.6
	Mutant	1 (11)	1 (33)	0 (0)	
BRAF status, *N* = 9(%)	Wild	8 (89)	2 (67)	6 (100)	0.6
	Mutant	1 (11)	1 (33)	0 (0)	
Disease-free survival	Median (months)	30	43	28	0.01
	1 year (%)	89	93	86	
	3 years (%)	35	61	24

### Univariate and multivariate outcomes

Univariate and multivariate analysis results are summarized in Table 3. Grade (poorly vs well-intermediate), stage groups (0–2 vs 3), lymph nodes (N0 vs N1–2), microsatellite instability (low vs high), and tumor budding (low-intermediate vs high) had a significant statistical association with DFS in univariate analysis. In multivariate analysis, grade and tumor budding status were found to be independent prognostic factors for DFS (*p* = 0.00).

**Table 3:** Cox-regression model of disease-free survival (DFS) in rectum cancer

	*n*	Median DFS (months)	Univariate analysis				Multivariate analysis			
			HR	95% CI		*p*	HR	95% CI		*p*
				Lower	Upper			Lower	Upper	
Gender	Female (*n* = 24)	28	0.69	0.35	1.36	0.29				
	Male (*n* = 51)	31								
Grade	Poorly (*n* = 20)	22	2.74	1.45	5.19	0.00	3,37	1,75	6.49	0.00
	Well-intermediate (*n* = 55)	32								
Stage	Stage 1–2 (*n* = 39)	38	2.54	1.31	4.90	0.00				
	Stage 3 (*n* = 36)	28								
Lymph nodes	N 0 (*n* = 39)	42	2.37	1.22	4.59	0.01				
	N 1–2 (*n* = 36)	28								
Kras status, *n* = 41	Wild (*n* = 9)	23	1.42	0.71	3.72	0.2				
	Mutant (*n* = 32)	24								
Microsatellite instability status	Low (*n* = 48)	38	2.44	1.31	4.55	0.00				
	High (*n* = 27)	26								
Tumor budding	Low-intermediate (*n* = 29)	43	2.57	1.18	5.57	0.01	3.14	1.42	6.94	0.00
	High (*n* = 46)	28								

## Discussion

Our study is a report on high tumor budding status relationship of survival. We demonstrated that high tumor budding was correlated with poor disease-free survival and found independent prognostic factor by multivariate analysis.

In the recent years, several trials showed that tumor budding associated with different clinical and histological parameters such as tumor site, tumor size, histologic type, grade, nodal involvement, AJCC stage, distant metastasis, and local recurrence. Ohtsuki et al. evaluated 149 patients with colorectal cancer and reported that tumor budding significantly associated with wall penetration, the incidence of lymph node metastasis, and cancer recurrence [18]. Another study reported that high budding associated with an infiltrative growth pattern and lymphovascular invasion. Five-year cancer-specific survival was significantly poorer in high compared with low budding groups [19]. Sevda et al. showed a statistically significant relationship between high-grade tumor budding density and histological grade, lymph node involvement, and vascular invasion in their research [20]. Conservely, in our study, we did not find an association between tumor budding, histological grade, and lymph node involvement. We evaluated only patients with treated neoadjuvant chemoradiotherapy which situation may have affected our assessment of tumor grade and lymph node involvement.

There are several studies in the literature investigating the relationship between tumor budding and disease-free survival. In a study of 138 patients diagnosed with stage 2 colon cancer, Tanaka et al. reported that the cumulative disease-specific survival rates at 5 years for patients with tumor budding low and high were 98 and 74%, respectively [21]. As mentioned above, Wang et al. also showed that the 5-year cancer-specific survival was significantly poorer in high compared with low budding groups [19]. Petrelli et al., in their meta-analysis, showed that tumor budding was a significant histologic marker in the prognosis of stage 2 colorectal cancer and high-grade tumor budding in these patients is related to a 25% increase at the risk of death in 5 years. Researchers suggested that this may be a useful prognostic factor when deciding the adjuvant treatment in these patients groups [22]. Another systematic review and meta-analysis conducted in 2016 have confirmed the impact of tumor budding in colorectal cancer, and it can be a predictive marker of recurrence and cancer-related death at 5 years [23]. Also, Cappellesso et al., in their meta-analysis which included 41 studies involving a total of 10,137 patients, demonstrated the prognostic value of tumor budding in pT1 colorectal cancer patients. Nodal metastasis was 28.5% in tumor budding positive and 7.2% in negative patients. Researchers revealed that tumor budding positivity increased the risk of nodal metastasis by 6.44 (OR value of 6.44 (95% CI 5.26–7.87; *p* < 0.0001; *I*^2^ = 30%; 41 studies) and concluded it was an independent histologic prognostic biomarker in pT1 colorectal cancer [24].

Jager et al. investigated the prognostic value of tumor budding for neoadjuvant treatment response in a cohort of 128 rectum cancer patients. Positive tumor budding was associated with significantly reduced T-level downstaging, tumor regression, and poor 5-year relapse-free survival. Besides, a multivariate analysis confirmed a positive tumor budding after neoadjuvant chemoradiotherapy as a negative predictive histologic biomarker for relapse-free survival [25]. In another study conducted in 2012 in which the same group of patients was included, tumor budding was found to be an independent prognostic factor in terms of disease-free survival [26]. Tumor regression especially pathological complete response following neoadjuvant treatment is related to long-term survival with low rates of local recurrence and distant metastasis [27]. Therefore, a standardized pathological evaluation after chemoradiotherapy in rectal cancer is recommended. Tumor regression grade (TRG) has been defined as a useful method of scoring tumor response [28, 29]. Fokas et al. reported that higher TRG after neoadjuvant chemoradiotherapy predicted a favorable long-term outcome [30]. In our study, the relationship between tumor budding and disease-free survival is similar to the literature. We found a median DFS of 43 months in tumor budding low-intermediate groups and 28 months in high groups. One and three-year disease-free survival rates were higher in the tumor budding low-intermediate group compared to the high group. High tumor budding in multivariate analysis was found as an independent prognostic factor for disease-free survival. On the other hand, we did not find any significant association with tumor response, lymph node involvement, and grade between two groups. Unlike Jager et al., this situation may be related to the different groupings according to tumor budding in our study. In conclusion, further phase 3 trials are needed to validate TRG and tumor budding (or related with together use) as a surrogate marker for survival in rectum cancer patients after neoadjuvant treatment. The current literature which is investigating the prognostic role of tumor budding in patients with rectum cancer who underwent neoadjuvant chemoradiotherapy was outlined in Table 4.

**Table 4:** Review of current literature investigating tumor budding in a rectum cancer patient who was treated with neoadjuvant chemoradiotherapy

		Due et al. [26].	Jager et al. [25].	Our study
Design		Retrospective	Retrospective	Retrospective
Follow-up period		2001–2005	2003–2012	2013–2018
Patients (*n*)		96	128	75
Neoadjuvant protocols	Radiotherapy	3000 cGy in 10 fractions in 2 weeks	45–50 Gy 5–6 weeks	45 Gy 4 weeks
	Concurrent chemotherapy	Absent	5-Flouracil, capecitabine, oxaliplatin	5- Flouracil, capecitabine,
Interval to surgery (weeks)		2–3	3–9	8–12
Postoperative treatment (*n*)		All patients	58 patients	All patients
Median follow-up (months)		70.8	84	35
Tumor budding		0–9 buds: low grade 10 or more buds: high grade	0 buds: none 1 bud: mild 2–4 buds: moderate 5 or more buds: severe	0–4 buds: low budding 5–9 buds: intermediate budding 10 or more buds: high budding
Association with		Disease-free survival	Relapse-free survival, distant and overall recurrence	Disease-free survival
Result		Low vs high	None-mild vs moderate-severe	Low-intermediate vs high
		5-year DFS, 87.5% vs 55.6%	5-year RFS, 90% vs %71%	3-year DFS, 61% vs 24%

Tumor budding has also evaluated in other gastrointestinal cancers such as squamous esophageal cancer, gastric adenocarcinoma, and pancreatic cancer patients [31–33]. High tumor budding in the three cancers has also shown to be associated with poor prognosis. Additionally, several non-gastrointestinal cancers such as lung cancer, head and neck carcinomas, and breast cancer have shown that tumor budding was a prognostic factor [34–36].

## Conclusion

We had some limitations in our study. Firstly, the relatively low number of patients may cause selection bias. Secondly, we also had to divide the patients into two groups. Therefore, we could not evaluate the relationship between the low and intermediate budding group clearly. Patients with high tumor budding have shorter disease-free survival than patients with low-intermediate tumor budding. In our patient population, having high tumor budding suggests a high likelihood of relapse. Therefore, we might need additional follow-up protocol in these patients.
